# Childhood Conditions Influence Adult Progesterone Levels

**DOI:** 10.1371/journal.pmed.0040167

**Published:** 2007-05-15

**Authors:** Alejandra Núñez-de la Mora, Robert T Chatterton, Osul A Choudhury, Dora A Napolitano, Gillian R Bentley

**Affiliations:** 1 Department of Anthropology, University College London, London, United Kingdom; 2 Department of Obstetrics and Gynaecology, Northwestern University, Chicago, Illinois, United States of America; 3 Department of Microbiology, Sylhet Osmani Medical College, Sylhet, Bangladesh; Imperial College London, United Kingdom

## Abstract

**Background:**

Average profiles of salivary progesterone in women vary significantly at the inter- and intrapopulation level as a function of age and acute energetic conditions related to energy intake, energy expenditure, or a combination of both. In addition to acute stressors, baseline progesterone levels differ among populations. The causes of such chronic differences are not well understood, but it has been hypothesised that they may result from varying tempos of growth and maturation and, by implication, from diverse environmental conditions encountered during childhood and adolescence.

**Methods and Findings:**

To test this hypothesis, we conducted a migrant study among first- and second-generation Bangladeshi women aged 19–39 who migrated to London, UK at different points in the life-course, women still resident in Bangladesh, and women of European descent living in neighbourhoods similar to those of the migrants in London (total *n* = 227). Data collected included saliva samples for radioimmunoassay of progesterone, anthropometrics, and information from questionnaires on diet, lifestyle, and health. Results from multiple linear regression, controlled for anthropometric and reproductive variables, show that women who spend their childhood in conditions of low energy expenditure, stable energy intake, good sanitation, low immune challenges, and good health care in the UK have up to 103% higher levels of salivary progesterone and an earlier maturation than women who develop in less optimal conditions in Sylhet, Bangladesh (*F*_9,178_ = 5.05, *p* < 0.001, standard error of the mean = 0.32; adjusted *R* ^2^ = 0.16). Our results point to the period prior to puberty as a sensitive phase when changes in environmental conditions positively impact developmental tempos such as menarcheal age (*F*_2,81_ = 3.21, *p* = 0.03) and patterns of ovarian function as measured using salivary progesterone (*F*_2,81_ = 3.14, *p* = 0.04).

**Conclusions:**

This research demonstrates that human females use an extended period of the life cycle prior to reproductive maturation to monitor their environment and to modulate reproductive steroid levels in accordance with projected conditions they might encounter as adults. Given the prolonged investment of human pregnancy and lactation, such plasticity (extending beyond any intrauterine programming) enables a more flexible and finely tuned adjustment to the potential constraints or opportunities of the later adult environment. This research is the first, to our knowledge, to demonstrate a postuterine developmental component to variation in reproductive steroid levels in women.

## Abstract 2

Alejandra Núñez-de la Mora and colleagues found that women of Bangladeshi origin who had spent their childhood in the UK had higher progesterone levels and matured earlier than those who had been children in Bangladesh.

## Editors' Summary

**Background.:**

Women's reproductive biology—which includes levels of sex hormones at different stages of the menstrual cycle—is known to vary greatly depending on the environment. This variation can be measured by looking at the levels of certain reproductive hormones in the saliva, for example, progesterone and estradiol. The levels of these hormones differ greatly from individual to individual. Women who live in environments where less food is available or infections are more common, or who do more physical work seem to have lower levels of these hormones compared to other women. Because these differences seem to persist over the long term, some researchers have suggested that various factors relating to an individual's environment early in life have a strong influence on later fertility. This theory is based on ideas drawn from evolutionary biology and ecology. Theoretically, a woman who limits her future fertility as a biological response to poor environments would eventually have greater evolutionary success than an individual who remains fertile in harsh conditions. Although the logical basis for this idea is attractive, there is not very much evidence that it is true.

**Why Was This Study Done?:**

In this study, the researchers wanted to collect experimental evidence from human populations that would help them test the hypothesis outlined above. The study performed here made use of a “natural experiment” resulting from widespread migration of people from Bangladesh to the UK. 95% of these individuals come from one particular region of Bangladesh, Sylhet. People would experience extensive differences between the environments before and after migration, including economic factors, access to health services, exposure to infectious diseases, and so on. Therefore, a comparison of hormone levels between individuals who had migrated at different periods during their life might help to test the theory that early environmental factors affect later fertility.

**What Did the Researchers Do and Find?:**

Five groups of women were selected and compared in this study. The groups included women who had grown up in Bangladesh but moved to the UK as adults; women who were born in Bangladesh but moved to the UK as children; women whose parents were from Bangladesh but who were born and raised in the UK; Bangladeshi women who were born and raised in Bangladesh; and finally a comparison group of women of European descent who were born and raised in the UK. Each woman in each of the five groups was asked to collect a sample of her own saliva each day for one menstrual cycle. The researchers tested the saliva samples to find out the levels of progesterone. The average progesterone level for the last 14 days of each woman's menstrual cycle (when progesterone levels are generally high) was then calculated and these values compared across the five groups. In particular, the researchers looked at the relationship between the age at which an individual arrived in the UK, the time spent in the UK, and the progesterone levels in saliva.

The researchers found statistically significant differences in progesterone levels between individuals who had migrated as children, second-generation migrants, and women of European descent, as compared to the levels for Bangladeshi women who had never migrated and women who migrated as adults. Progesterone levels for the first three groups were higher than those for the Bangladeshi women who had not migrated or who had migrated as adults. The age at which women migrated also seemed to be linked to their progesterone levels. Amongst women who migrated before the start of menstruation, those who migrated at a younger age had higher average progesterone levels. However, this relationship did not seem to hold true for women who had migrated after they started menstruating.

**What Do These Findings Mean?:**

This study suggests that certain indicators of reproductive biology, such as progesterone levels, may be linked to environmental factors that an individual experiences early in life. The findings support the idea that harsh environments early in development are associated with lower fertility later in life. However, it is not clear from this study which precise environmental factors are involved; it is possible that exposure to infectious disease is important, but this possibility was not specifically tested in this research study. Finally, it is not certain whether the lower average progesterone levels of the particular groups studied would actually translate into lower fertility. It is possible that although certain individuals and groups had lower levels of this hormone, they may in fact have been as fertile as individuals with higher levels of the hormone.

**Additional Information.:**

Please access these Web sites via the online version of this summary at http://dx.doi.org/10.1371/journal.pmed.0040167.

- Information from the US National Institutes of Health on fertility and infertility
- Wikipedia entry on progesterone (Wikipedia is an internet encyclopedia anyone can edit)
- Resources from the World Health Organization about sexual and reproductive health, including links to resources on family planning, infertility, and other related topics
- World Health Organization country site for Bangladesh, providing key statistics and health resources
- Moving Here: stories and experiences told of individuals' emigration to the UK

## Introduction

For the past two decades, innovative methods for monitoring ovarian function have encouraged the detailed study of human reproductive function among genetically, culturally, and ecologically diverse populations in field settings. Research by reproductive ecologists has established that levels of ovarian function, measured as average profiles of salivary progesterone and oestradiol, differ considerably within and between individuals and populations [1,2]. Detailed studies have demonstrated that age [3] and acute energetic factors such as immune challenges [4], nutrition and/or energy expenditure, whether voluntarily imposed [5,6] or ecologically determined [7–9], are responsible for some of this variance. However, even after acute factors are accounted for, significant interpopulation differences in baseline levels of ovarian function remain evident. Energetically, nutritionally, or immunologically challenged populations exhibit chronically lower profiles of reproductive steroids than their affluent counterparts. The question of how this variation arises and what its functional significance may be is still poorly understood.

Based on indirect evidence for an association between chronic energy availability and age at menarche (proxy for maturation tempo) [10–12], and empirical data on the inverse relationship between menarcheal age and levels of ovarian function [13–16], researchers have postulated that baseline variation in ovarian function may have a developmental origin [17,18]. It has been proposed that variation in adult levels of reproductive hormones occurs as a consequence of differences in chronic energy availability during growth and development which, in turn, result in diverse maturation tempos and the establishment of different adult physiological set points for regulation of the hypothalamic–pituitary–ovarian and related metabolic axes [18,19]. This hypothesis sits within the framework of life history theory, which posits that energy, as a limited resource, needs to be allocated between maintenance, growth, and reproductive functions, thus generating trade-offs. The way in which such trade-offs are resolved is driven by natural selection to yield optimal allocation patterns within specific relevant constraints [20]. In this context, the rates of childhood growth and adolescent maturation provide a bioassay of environmental quality, particularly the overall level of energy above maintenance costs that is likely to be available for reproduction in later life [18].

The developmental hypothesis therefore predicts that adverse energetic conditions during development that result in slow growth and delayed reproductive maturation lead to lower baseline levels of ovarian function and heightened ovarian sensitivity to ecological factors throughout life. Since lower levels of ovarian hormones are associated with a reduced probability of conception and implantation [21,22] and increased average inter-birth intervals [23], such a conservative reproductive strategy would optimise lifetime resource allocation, enhance survival, and ultimately increase fitness [18].

Although there is strong theoretical support for the developmental origin of interpopulation variation in ovarian function, empirical evidence is circumstantial. Here, we use a migrant study as a natural experiment to evaluate the effects of environmental conditions on adult levels of ovarian hormonal function, and to assess whether there are any critical windows during development, when changes in such conditions may have significant effects. The study compares groups of women with similar genetic background who, as a result of migration, spent their formative years in contrasting environments; namely, Sylhet, Bangladesh (lower living standards), and London, England (higher living standards). Saliva samples were collected for analyses of both salivary progesterone and oestradiol. We report here on results for salivary progesterone and the implications of these results for reproductive function within the context of both life history theory and for changing health risks for populations of migrants to developed industrialised countries. Results for salivary oestradiol are reported elsewhere [24,25].

In this study, we therefore tested the following hypotheses and predictions.

(A) Early life conditions will influence adult baseline levels of progesterone. Prediction A: Women who migrated from Bangladesh to the UK as children, second-generation UK-Bangladeshis, and women of European descent will have higher progesterone levels than Bangladeshi sedentees.

(B) A positive change in environmental conditions will impact developmental tempo and will be reflected in enhanced reproductive hormonal function. Prediction B: Women who migrated as children will have an earlier maturation and higher mean progesterone levels than women who migrated as adults.

(C) Alterations in conditions after maturation will not modify hormonal set points established during development. Prediction C: Adult migrants will have baseline progesterone levels that are comparable to Bangladeshi sedentees despite differences in current environmental conditions.

## Methods

### Study Population

Bangladeshi migrants in London were selected as a study population because they originate from one of the poorest nations in the world with economic, developmental, and health indicators that differ substantially from those of the UK [26,27], accentuating the contrast between the pre- and postmigration environments and improving study design. For instance, Bangladesh's per capita gross national product is only 1.4% that of the UK, life expectancy for women is 25% shorter, and the prevalence of low birth weight is four times higher than in the UK [26,27]. Ninety-five percent of Bangladeshi migrants originate from a single geographical region [28]—Sylhet District—and have low rates of intermarriage with other ethnic groups, thus reducing genetic “noise” [29]. Bangladeshis in the UK are socioeconomically homogeneous, share similar histories of migration [30,31], and constitute a multigenerational population that allows for recruitment of individuals who entered Britain at different life stages (infancy, childhood, and adulthood) [32,33].

Most Bangladeshi migrants originate from relatively affluent, land-owning classes in Sylhet who live in solid dwellings, have access to good nutrition, and have low levels of energy expenditure [28]. They are not, therefore, comparable to the rural, malnourished populations, such as those in Matlab thana studied by other researchers in Bangladesh (e.g., [34,35]). In addition, the diet of Sylheti migrants in London remains remarkably similar following migration due to the availability of a wide range of fresh and frozen produce from Bangladesh in local shops and supermarkets [36]. However, despite their relative affluence, Sylheti residents in Bangladesh still suffer from higher immune challenges due to poor sanitation, the effects of which are exacerbated by the limited availability and poor quality of health care provision [37–39]. The towns and villages in which participants live lack appropriate sewage and waste disposal systems, have limited water treatment capacity, and are subject to seasonal floods, resulting in an environment where infection and disease are common and potable water is scarce [40]. Thus, despite the fact that Bangladeshi migrants in the UK have socioeconomic indicators that reflect their socially disadvantaged position relative to the general British population [31], the overall good quality of the environment and access to health services, and a clean water supply in the UK, represent a radical departure from conditions in Sylhet. If nothing else, migrants to the UK are free from chronic exposure to infections [41]; this difference would be expected to translate into enhanced growth and maturation [42].

Estimates available from cross-sectional studies confirm this latter prediction. The growth of infants and children of South Asian background is comparable to the 1990 UK growth standards [43]. Similarly, data confirm a secular trend toward increased height in succeeding generations, especially in females [44]. With respect to infant and child health, a study in East London, where the concentration of Bangladeshi migrants is highest, revealed that post-neonatal mortality rates for infants born to Bengali mothers between 1987 and 1990 was 6.9 per 1,000 live births [45], one-tenth as high as the national rates of 56 per 1,000 births for Bangladesh [26]. These indicators support the argument that women in the present study are indeed likely to have experienced contrasting environmental conditions during development, depending on their country of birth and the place in which they spent their childhood and adolescence.

### Study Design

The effect of different formative environments on adult levels of ovarian function was examined by comparing salivary progesterone in five different groups—three migrant and two reference groups. Migrant groups included (A) first-generation migrants (*n* = 56) who spent their infancy and childhood in Bangladesh but moved to the UK as adults (postmenarche); (B) first-generation migrants (*n* = 42) born in Bangladesh but who moved to the UK as infants or children (premenarche); and (C) second-generation women (*n* = 33) conceived, born, and raised in the UK whose parents had originally migrated from Bangladesh. Reference groups contained (4) Bangladeshi women (*n* = 48) born, raised, and resident in Bangladesh (sedentees); and (5) women of European descent (*n* = 48) living in London neighbourhoods similar to those of the Bangladeshis, whose parents, and at least both grandmothers, were born in the UK.

### Recruitment Procedure

Sylhet District in northeastern Bangladesh was chosen as the target area for recruitment of Bangladeshi sedentees because over 95% of migrants to the UK originate from this region [28]. The majority of participants (91%) were from Sylhet town (285,300 inhabitants), the biggest settlement in the District, with fewer subjects from the villages of Barokote and Uttar Royghar in Golapganj thana (about 15 km southeast of Sylhet town). All women were contacted through networking and word of mouth by a team of bilingual (Sylheti–English) research assistants.

In London, Bangladeshi women were contacted by bilingual workers at local schools, community centres, mosques, and sports centres in the boroughs of Tower Hamlets (East London) and Camden, where the London population of Bangladeshis is primarily concentrated [46]. Women of European descent were recruited mainly through advertisements in local Camden and East London newspapers.

### Participants

Participants were well nourished, healthy, regularly menstruating women (i.e., never or rarely missing a menses and menstrual cycles between 23 and 37 d in length) of reproductive age (19–39 y), with no clinical history of diabetes, infertility, thyroid, or reproductive disorders, and who were not currently (or for the past 3 mo) pregnant, lactating, or using steroid-based contraceptives. These criteria were designed to screen out women whose steroid levels would be altered by certain pathologies, reproductive conditions, or exogenous hormones. Except for the occasional short visit to Bangladesh, all first-generation migrant women had lived in the UK uninterruptedly since arrival. Similarly, Sylheti, second-generation Bangladeshi women and women of European descent had lived continuously in Bangladesh or the UK, respectively. None of the participants was genetically related.

### Sample Collection and Hormonal Analyses

A pilot study was first conducted to assess potential contamination of saliva samples from chewing betel nut [47], a common practice among Bangladeshis in the UK [48]. Collection protocols were then adjusted to ensure that betel chewers waited at least 1 h after chewing before expectoration. Women were similarly asked not to eat, drink (other than water), or brush their teeth for 1 h prior to sample collection, to avoid sample contamination. Participants collected daily saliva samples for the duration of a menstrual cycle starting on the first day of menses. Women were encouraged to collect samples at roughly the same time of day. These consisted of 5 ml of saliva collected in polystyrene tubes pretreated with sodium azide as a preservative to a final concentration of approximately 0.1%. A sugarless spearmint-flavoured gum was used as salivary stimulant. This gum has been extensively tested and does not interfere with analyses of salivary steroids [49]. Saliva was assayed for progesterone using standardised radioimmunoassay techniques [49]. Intra- and interassay coefficient variations were 11.6% and 14.7%, respectively. Assay sensitivity was 15 pg/ml. All data are presented in pg/ml.

At the end of the menstrual cycle for which saliva samples were collected, anthropometric measurements (height, weight, and skinfolds) were taken following standard procedures [50] and two questionnaires (general sociodemographic and food frequency) were administered on a one-to-one basis in either English or Bengali by a bilingual female researcher. A small proportion (15%) of participants in the European-descent group completed their own questionnaires and returned them to the researchers by surface mail. These data were used to make intergroup comparisons and to evaluate changes in standards of living, diet, health, reproductive patterns, and lifestyle sequential to the migration experience.

### Statistical Analyses

Mean progesterone values for the last 14 d of the menstrual cycle (luteal phase) were calculated as a quantitative index of ovarian function following standard procedures [17]. Individual luteal progesterone indices were pooled, and averages per group calculated. Indices were log transformed to reduce positive skewness. Group differences in luteal progesterone indices and the effects of anthropometric and reproductive variables were evaluated by standard multiple linear regression models. Age, body mass index, height, and age at menarche were entered as independent variables using continuous and simple linear terms. “Group” was entered as a categorical variable using sedentees as the reference.

Pearson's correlation was used to examine the relationships between: (A) Age on arrival to the UK with age at menarche and steroid levels; (B) time spent in the UK and steroid levels among women who migrated after puberty; and (C) steroid levels with age at menarche. For the former analysis, all first generation (Bangladesh-born) women in the study were classified according to whether their age on arrival was younger or older than the maximum recorded age at menarche (16 y). Women who arrived in the UK at ages 16 y or younger were thus classified as “child” migrants, whereas those who entered the country at older ages (>16 y) were categorised as “adults.” Bangladeshi women who migrated prior to menarche were then divided into two groups according to age at migration: (0–8 y; *n* = 22) and (≥9 y; *n* = 17). The transition at 8 y was chosen to approximate the growth period prior to adrenarche, when critical endocrinological changes occur as a precursor to adolescent hormonal development [51]. Differences in luteal progesterone indices between these two categories were analysed by general linear models. Significance level was set at *p* < 0.05. Statistical analyses were performed using SPSS v. 10.0 (http://www.spss.com).

### Research Ethics

Research protocols were approved by the Ethics Committees of University College London Hospital, Camden; Islington Local Health Authority, East London; and the City Local Health Authority and Sylhet Osmani Medical College, Bangladesh. Written informed consent was obtained from all participants in the study. All data were collected and stored in compliance with the Data Protection Act, UK.

## Results

**Figure 1:**
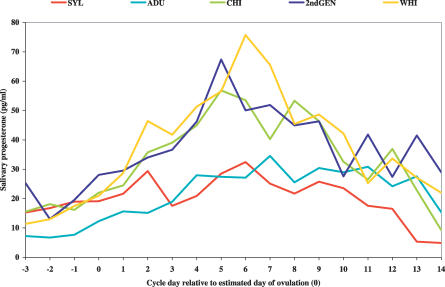
Average Luteal Progesterone Profiles by Group Unadjusted mean luteal progesterone index values. Mean ± SEM: 22.2 ± 3.1 pg/ml (resident Bangladeshi sedentees from Sylhet [SYL], *n* = 39); 24.6 ± 2.5 pg/ml (adult migrants [ADU], *n* = 46); 38.9 ± 5.7 pg/ml (child migrants [CHI], *n* = 39); 40.2 ± 5.5 pg/ml (second-generation British-Bangladeshis [2ndGEN], *n* = 29); 45.1 ± 4.3 pg/ml (British women of European descent [WHI], *n* = 45). Sample sizes include only the cycles for which an oestradiol midcycle peak and luteal progesterone rise were discernable (81%, 82%, 93%, 88%, and 94% of the original sample for SYL, ADU, CHI, 2ndGEN, and WHI groups, respectively). Oestradiol values were obtained from data available for the same individual menstrual cycles. Ovulation dates were estimated from individual oestradiol data [22]. Confidence intervals are omitted for visual clarity. Sample sizes include all women for which hormonal data were available and may differ from total sample sizes for other aspects of data collection.

Multiple linear regression models showed significant differences in luteal progesterone levels between groups (*F*_9,178_ = 5.05, *p* < 0.001, standard error of the mean [SEM] = 0.32; adjusted *R* ^2^ = 0.16) (Table 1). Average luteal progesterone values, controlling for anthropometric and reproductive variables, were significantly higher in the child migrant, second-generation, and European-descent groups (75%, 81%, and 103%, respectively) than those for sedentees. In contrast, no significant differences in average progesterone indices between adult migrants and sedentees were found (Figure 1; Table 1). Average steroid levels and maturation age were negatively correlated across groups (*r* = −0.17, *p* = 0.02, *n* = 198).

**Table 1:**
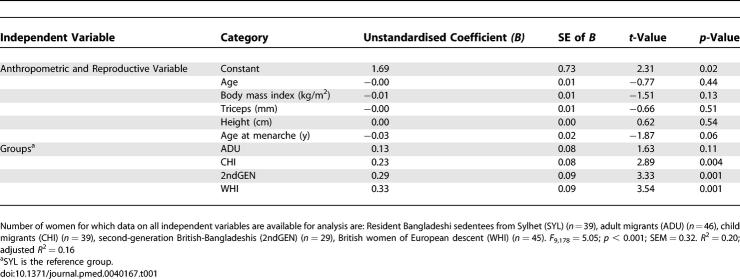
Multiple Linear Regression Model for Luteal Progesterone Index

**Figure 2:**
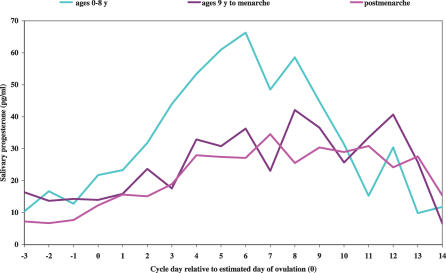
Average Luteal Progesterone Profiles by Categories of Age at Migration to the UK Unadjusted mean luteal progesterone index values. Mean ± SEM: 44.3 ± 8.6 pg/ml (ages 0–8 y, *n* = 22); 31.8 ± 6.8 pg/ml (ages 9 y to menarche, *n* = 17); 24.6 ± 2.5 pg/ml (postmenarche, *n* = 46). Ovulation dates were estimated from oestradiol data available for the same individual menstrual cycles [22]. Confidence intervals are omitted for visual clarity. Sample sizes include all women for which hormonal data were available and may differ from total sample size for other aspects of data collection.

For child migrants, age at migration was significantly correlated with menarcheal age (*r* = 0.31, *p* = 0.03, *n* = 39). Similarly, age at migration was negatively correlated with average luteal progesterone values among women who migrated prior to menarche (*r* = −0.28, *p* = 0.04, *n* =39), but was not significantly correlated among postmenarcheal (adult) migrants (*r* = 0.16, *p* = 0.33, *n* = 46). Furthermore, women who migrated during infancy and early childhood (ages 0–8 y) had a significantly earlier age at menarche (*F*_2,86_ = 3.21, *p* = 0.03), were significantly taller (*F*_2,88_ = 3.7, *p* = 0.01), and had, after controlling for age, anthropometric and reproductive variables, higher average luteal progesterone (*F*_2,81_ = 3.14, *p* = 0.04) than those who migrated at a later stage (including child migrants from age 9 y to menarche, and adult migrants) (Figure 2).

Finally, among women who migrated after menarche, length of time spent in the UK had no significant impact on luteal progesterone levels (*r* = −0.20, *p* = 0.18, *n* = 46).

## Discussion

To our knowledge this is the first study to show that adult reproductive steroid levels in women are influenced by experiences during a critical developmental phase in childhood prior to adolescence. Bangladeshi women who spent their childhood in Bangladesh—whether sedentees or adult migrants—had significantly lower levels of salivary progesterone and a slower maturational trajectory than did Bangladeshi women and women of European descent who grew up in London.

From a life history perspective it would be adaptive to adjust developmental trajectories to changing levels of energy that favour reproduction above basic maintenance costs. In this study, progesterone profiles as well as height and menarcheal age—both proxies of developmental tempo—respond positively to improved environmental conditions during childhood. These simultaneous effects suggest that growth, maturation, and ovarian function are linked to an overall programme of energy allocation moderated by assessing environmental impacts including nutrition, energy expenditure, and immunological stressors, and possibly mediated through metabolic hormones such as insulin and/or insulin-like growth factors operating either directly on the ovary, or via the hypothalamus, or both [19,52].

That the period of early postnatal life is critical for the establishment of growth and maturation trajectories has been identified in other studies [10,42]. The shift in regulatory and secretory patterns in various metabolic and endocrine axes during infancy, likely in coordination with neurological development, may mediate the observed effects of improved environmental quality on trajectories of development [53].

Ethnographic, anthropometric, and dietary data for the relatively affluent Bangladeshi women who participated in the study indicate that the taxing maintenance costs for the women who grew up in Sylhet, which result in lower levels of ovarian function, are not primarily linked to nutritional factors and/or heavy physical workloads as demonstrated in other populations [54]. Instead, the lower levels of salivary progesterone are likely related to the chronic immune challenges associated with high pathogen loads and infectious exposures [37,39,41].

Links between epidemiological factors and chronic energy availability have been widely documented. For instance, chronic morbidity has been shown to have a negative impact on growth and maturation [55–57], even in conditions of adequate nutrition [58]. Furthermore, analysis of historical data from four northern European countries has revealed an association between increased adult height and reduced old-age mortality related to a lifetime reduction of infection and inflammation [59]. Similarly, the secular trend towards earlier maturation in humans finds its closest correlate with the declining mortality rates of the demographic transition (underscored by a dramatic shift in patterns of morbidity) [60]. Together these findings fit the prediction of life history theory, namely that individuals developing in a high morbidity environment would have to trade off survival at high energetic costs (through the maintenance of a chronically stimulated immune function) for a slower developmental trajectory with restricted growth, late maturation, and, consequently, lower levels of ovarian function.

It is possible that lower levels of progesterone described here in the adult and child migrant groups might be related either to the stress of migration, or of participation in a project where saliva is collected and where there is a lack of familiarity with this procedure [61]. If this were the case, then we would expect levels of progesterone to be higher among adult migrants who have lived in the UK for longer periods of time (controlling for age). However, we do not observe such a trend in the data. Moreover, information collected from our extensive questionnaires, as well as discussions with many Bangladeshi migrants, reveal that child migrants and second-generation women are exposed to more daily stressors than adult migrants. The former two groups are caught between two cultures, having been raised in the UK but still remaining embedded within the Bangladeshi community. Most child migrants and second-generation women hold jobs but still have many household tasks to complete at home. Many are married to men who were raised in Bangladesh but migrated to the UK to be their spouse; this often leads to family tensions. In our study 26% of second-generation women and 19% of child migrants were the sole breadwinners for the family, where their first-generation husbands might not have either the language or technical skills to obtain and retain viable employment in the UK. Again, given these stresses, we would expect that child migrants and second-generation women would have lower, rather than the observed higher levels of progesterone compared to adult migrants who are not exposed to these cultural tensions and work-related stresses [62].

It is difficult to determine how lower levels of progesterone might translate into differences in fecundity and fertility. In a study undertaken in rural highland Bolivia and Chicago that compared the fecundity of Aymara and US women, the former were able to conceive and maintain pregnancies at significantly lower levels of salivary progesterone than the latter [21,63]. However, there remain substantial differences in the reproductive status and behaviour of these two groups of women. Specifically, the Aymara are effectively a natural fertility population with negligible rates of contraceptive use; many women in this particular study conceived following periods of lactational amenorrhoea subsequent to prior pregnancies. This is in sharp contrast to the US women from Chicago, who were actively planning pregnancies and often using behavioural strategies to optimise the possibility of conception (such as timing of intercourse, ovulation predictor kits, and so forth).

In another study conducted among US women of European descent attempting to conceive, intra- and interindividual comparisons between naturally occurring conception and exposed nonconception cycles revealed that midfollicular salivary oestradiol levels were highly correlated with the probability of successful conception [22]. Similarly, salivary progesterone levels during the late midluteal phase (the presumed time of implantation) were significantly higher in conception cycles than in exposed nonconception ones [22,64]. Similar results were obtained for urinary oestrogen and progesterone metabolites among Chinese women [65]. These findings suggest that fecundability (the monthly probability of conception) would be lower among women with generally lower average levels of salivary progesterone. Lower fecundability in cycles with lower reproductive steroid levels would certainly be in accordance with expectations from life history theory. To test whether these expectations apply to the Bangladeshi population here would require further study of reproductive steroid levels during both conception and nonconception cycles.

The critical developmental phase in which improved conditions affect adult progesterone levels in our study of Bangladeshi migrants occurs prior to age 8 y. As mentioned earlier, adrenarche occurs at approximately this age, is associated with the onset of adrenal androgen production presaging puberty, and is a process significant in the timing of reproductive maturation [53]. In essence, this period may represent a last “checkpoint” for women at which to assess environmental conditions and adjust developmental trajectories accordingly. Further work on adrenal androgens and ovarian steroid hormones during childhood and adolescence in Bangladeshi migrants and sedentees will shed more light on the importance of this phase for later reproductive development.

Recent research has underscored the utility of a life history perspective in epidemiological studies [66]. The findings reported here of a postuterine, childhood phase during which adult set points of salivary progesterone can be affected adds a new dimension to the study of the aetiology and epidemiology of women's reproductive health in later life. In addition, these results are relevant in assessing differential potential health risks associated with ovarian steroid hormone production that might be sequential to the migration experience.

One such health risk is the development of breast cancer. Ovarian steroid hormones have been identified as important factors in the development and prognosis of this pathology [67,68]. At the cellular level, both oestradiol and progesterone act as potent mitogens in normal and neoplastic breast tissue [69]. At an epidemiological level, although the evidence for an association between high levels of progesterone and a high risk of breast cancer is less consistent than it is for oestrogens, there are correlational data that point to a strong positive relationship between average progesterone values and breast cancer incidence rates [70]. Thus, we suggest that the significant increase in chronic progesterone levels among migrant women documented here may result in higher breast cancer risk in subsequent generations of this community. This projection is also substantiated by a number of other physiological and behavioural changes that occur among South Asian women consequent to migration including an earlier menarche, earlier age at first birth, and a dramatic reduction in breast-feeding practices [62,71].

For South Asian women (a group that includes Indians, Pakistanis, and Bangladeshis) in the UK, there is a series of epidemiological studies that already document a transition in breast cancer incidence profiles. For example, a recent study in Leicester, a city with a large South Asian community, found that breast cancer rates among South Asians have increased over the last ten years while they have decreased in the non-Asian population [72]. Moreover, there is evidence to suggest that the increased risk for breast cancer is currently more marked for those age groups that include a substantial proportion of women actually born in the UK or who migrated during childhood [73]. Our data on generational trends in hormonal and developmental risk factors among precisely these migrant categories are congruent with these age cohort differentials in breast cancer incidence rates observed at the epidemiological level.

These findings add to accumulating evidence that humans have an evolved capacity to respond to chronic environmental conditions during growth that optimise resource allocation. This optimisation, however, may lead to specific developmental trajectories that compromise health in later life [74]. We show here that environmental conditions conducive to enhanced growth and maturation correlate with a suite of anthropometric, endocrine, and reproductive characteristics associated with high set points of ovarian function that could ultimately increase breast cancer risk. These results are pertinent not only to the Bangladeshi group we studied, but to many other migrant groups and populations in transition due to globalising forces. We believe, therefore, that in the face of increasing rural to urban and international migration worldwide, our reproductive hormonal findings could have potentially broad public health implications.

## Supporting Information

Alternative Language Abstract S1 Abstract translated into Spanish by Alejandra Núñez-de la Mora (27 KB DOC)

Alternative Language Abstract S2 Abstract translated into French by Verena Rubio-Godoy (27 KB DOC)

## Abbreviations

SEM: standard error of the mean
