# Supplementary material for: Childhood Conditions Influence Adult Progesterone Levels
Source: PLoS Med. 2007 May 15;4(5):e167. doi: 10.1371/journal.pmed.0040167 (PMC1868040; doi:10.1371/journal.pmed.0040167)
Supplement: Supplementary file 1 [file pmed.0040167.sd001.doc]

Las condiciones durante la infancia influyen en los niveles adultos de progesterona

Alejandra Núñez-de la Mora^1*^, Robert T. Chatterton^2^, Osul A. Choudhury^3^, Dora A. Napolitano^1^, Gillian R. Bentley^1^.

^1^ Departamento de Antropología, University College London, Londres, Reino Unido

^2^ Departamento de Obstetricia y Ginecología, Northwestern University, Chicago, EUA

^3^ Departamento de Microbiología, Sylhet Osmani Medical College, Sylhet, Bangladesh

Resumen

Antecedentes

En mujeres, los niveles promedio de progesterona en saliva varían signficativamente a nivel inter- e intra-poblacional en función de la edad y de las condiciones energéticas agudas relacionadas con la ingesta energética, el gasto energético o la combinación de ambos. Además de estos efectos agudos, existen también diferencias entre poblaciones respecto a los niveles *basales* de progesterona. Las causas de dichas diferencias crónicas no se comprenden del todo, pero se ha sugerido que pudieran resultar de distintos ritmos de crecimiento y maduración, y por ende, de las diversas condiciones medioambientales experimentadas durante la infancia y la adolescencia.

Metodología y resultados

Para probar dicha hipótesis, realizamos un estudio de migrantes con mujeres originarias de Bangladesh de primera y segunda generación, en edad reproductiva (19-39 años de edad), que migraron a Londres, Reino Unido en distintas fases del ciclo de vida, contrastándolas con mujeres residentes en Bangladesh y mujeres blancas que viven en barrios similares a los de las mujeres migrantes en Londres (total n=277). Se colectaron muestras de saliva para el análisis de los niveles de progesterona mediante la técnica de radioinmunoensayo, así como medidas antropométricas e información sobre dieta, estilos de vida y salud.

Los resultados de los análisis de regresión múltiple, tras corregir por variables antropométricas y reproductivas, muestran que las mujeres que pasaron su infancia en condiciones de bajo gasto energético, ingesta energética estable, buena sanidad, bajos retos inmunológicos y buen acceso a atención médica en el Reino Unido, tienen niveles de progesterona en saliva significativamente más altos (hasta un 103% más) y una maduración más temprana que las mujeres que se desarrollaron en condiciones menos favorables en Sylhet, Bangladesh (F _9,178_= 5.05, p< 0.001. s.e.m.= 0.32; R^2^ adj =0.16). Nuestros resultados apuntan a que el periodo previo a la pubertad es una fase sensible en la que los cambios en las condiciones medioambientales afectan positivamente el ritmo de desarrollo, tal como la edad a la menarquia (F _2,81_= 3.21, p= 0.03) y los patrones de función ovárica evaluados mediante niveles promedio de progesterona en saliva (F _2,81_= 3.14, p= 0.04).

Conclusiones

Este trabajo demuestra que las mujeres utilizan un periodo extenso del ciclo de vida previo a la madurez reproductiva para monitorear el medioambiente y modular los niveles de esteroides reproductivos en consecuencia. Dada la prolongada inversión de las mujeres de nuestra especie en el embarazo y la lactancia, dicha plasticidad (que se extiende más allá de cualquier programación intrauterina), permite un ajuste más flexible y mejor sincronizado a las potenciales limitaciones u oportunidades del medio ambiente previstas para la adultez. Esta investigación es la primera en demostrar un componente del desarrollo post-uterino en la varianza de los niveles de esteroides reproductivos en mujeres.

Translated into Spanish by: Alejandra Núñez-de la Mora
