# Supplementary material for: Childhood Conditions Influence Adult Progesterone Levels
Source: PLoS Med. 2007 May 15;4(5):e167. doi: 10.1371/journal.pmed.0040167 (PMC1868040; doi:10.1371/journal.pmed.0040167)
Supplement: Supplementary file 2 [file pmed.0040167.sd002.doc]

**Les conditions de vie enfantine influencent les niveaux de progestérone chez les adultes.**

Alejandra Núñez-de la Mora^1*^, Robert T. Chatterton^2^, Osul A. Choudhury^3^, Dora A. Napolitano^1^, Gillian R. Bentley^1^.

^1^ Département de Anthropologie, University College London, Londres, Royaume Uni

^2^ Département de Gynécologie Obstétrique, Northwestern University, Chicago, USA

^3^ Département de Microbiologie, Sylhet Osmani Medical College, Sylhet, Bangladesh

**Résumé**

Antécédents

Les profiles moyens de progestérone salivaire chez les femmes varient significativement, tant au niveau inter-, qu'au niveau intra-populaire, ceci en fonction de l'âge et des conditions énergétiques aiguës dépendantes de l'apport énergétique, de la dépense énergétique où de la combinaison de ces deux dernières. En plus des agents stressants aigus, les niveaux de base de progestérone sont différents parmi les populations. Les causes de tels différences chroniques ne sont pas tout à fait comprises, mais une hypothèse propose qu'elles peuvent être le résultat des variations dans le rythme de croissance et de maturation ainsi que, par implication, à l'exposition à différentes conditions environnementales pendant l'enfance et l'adolescence.

Méthodes et résultats

Pour tester cette hypothèse, nous avons fait un étude parmi trois groupes des femmes âgées entre 19 et 39 ans (n=227). Le première est formée par des femmes originaires du Bangladesh de première et deuxième génération qui sont arrivées à Londres, Royaume-Uni, à différents moments de leur cycle de vie. Le deuxième comprend des femmes habitant encore au Bangladesh et le dernier groupe comprend des femmes blanches habitant dans des quartiers similaires à ceux habités par les femmes émigrantes à Londres. Les données collectées incluent des échantillons de salive pour des essais radioimmunologiques de la progestérone, des mesures anthropométriques et des informations sur le régime alimentaire, le mode de vie et la santé.

Les résultats de régressions linéaires multiples effectuées pour contrôler des variations anthropométriques et reproductives ont montrés que les femmes au Royaume-Uni qui ont eu une enfance caractérisée par une dépense énergétique basse, une ingestion stable d'énergie, une bonne condition sanitaire, une exposition basse à des immunogènes et une bonne attention médicale, ont des niveaux de progestérone dans la salive significativement supérieurs (103% plus) ainsi qu'une maturation plus précoce par rapport à des femmes qui se sont développées dans des conditions moins optimales à Sylhet, Bangladesh (F _9,178_= 5.05, p< 0.001. s.e.m.= 0.32; R^2^ adj =0.16). Nos résultats indiquent que la période avant la puberté est une période sensible pendant laquelle des changements dans les conditions environnementales ont un impact sur le rythme de développement (F _2,81_= 3.21, p= 0.03) et sur les modèles de fonction des ovaires (F _2,81_= 3.14, p= 0.04).

Conclusion

Cette étude démontre que les femmes utilisent une grande période avant leur maturité sexuelle pour étudier leur environnement et moduler leurs niveaux des stéroides reproductifs en accord avec les possibles conditions de vie d'adulte. Etant donné que la grossesse humaine et la lactation représentent un investissement important, cette plasticité (qui s'étend au delà d'une programmation intra-utérine) permet un ajustement plus flexible et précis aux limitations ou opportunités potentielles de l'environnement adulte. Cette étude est la première à démontrer un component du développement post-utérine dans la variation des niveaux de stéroïdes reproductifs chez les femmes.

Translated into french by: Dr. Verena Rubio-Godoy
